# Oncologist burnout and compassion fatigue: investigating time pressure at work as a predictor and the mediating role of work-family conflict

**DOI:** 10.1186/s12913-017-2581-9

**Published:** 2017-09-11

**Authors:** Sibyl Kleiner, Jean E. Wallace

**Affiliations:** Department of Sociology, The University of Calgary, 2500 University Dr. N.W, Calgary, AB T2N 1N4 Canada

**Keywords:** Burnout, Compassion fatigue, Physicians, Mental health, Workload, Time pressure, Oncology, Cancer

## Abstract

**Background:**

Oncologists are at high risk of poor mental health. Prior research has focused on burnout, and has identified heavy workload as a key predictor. Compassion fatigue among physicians has generally received less attention, although medical specialties such as oncology may be especially at risk of compassion fatigue. We contribute to research by identifying predictors of both burnout and compassion fatigue among oncologists. In doing so, we distinguish between quantitative workload (e.g., work hours) and subjective work pressure, and test whether work-family conflict mediates the relationships between work pressure and burnout or compassion fatigue.

**Methods:**

In a cross-sectional study, oncologists from across Canada (*n* = 312) completed questionnaires assessing burnout, compassion fatigue, workload, time pressure at work, work-family conflict, and other personal, family, and occupational characteristics. Analyses use Ordinary Least Squares regression.

**Results:**

Subjective time pressure at work is a key predictor of both burnout and compassion fatigue. Our results also show that work-family conflict fully mediates these relationships. Overall, the models explain more of the variation in burnout as compared to compassion fatigue.

**Conclusions:**

Our study highlights the need to consider oncologists’ subjective time pressure, in addition to quantitative workload, in interventions to improve mental health. The findings also highlight a need to better understand additional predictors of compassion fatigue.

**Electronic supplementary material:**

The online version of this article (10.1186/s12913-017-2581-9) contains supplementary material, which is available to authorized users.

## Background

Physician suicide rates cross-nationally are elevated relative to the general population [1], with preventable work-related mental health problems such as burnout playing a key role in suicidal ideation [2, 3]. Suboptimal physician mental health is also tied to poorer quality of patient care and greater risk of medical errors [4]. We focus on both burnout, which has been shown to be high among physicians and on the rise in recent years [5], and compassion fatigue, which is rarely studied among physicians, but understood to be a consequence of caring for the suffering.

Oncologists are particularly at risk for mental health problems due to the emotionally demanding nature of their work [6]. Oncologists report higher levels of burnout than other cancer care staff [7], and the oncology specialty has been rated by physicians as a field producing “high” levels of emotional exhaustion [8]. Oncologists face the stress of heavy workloads [9] and patient loss as an “intrinsic” part of the job [10]. Oncology is therefore particularly suitable for examining the work-related factors associated with both burnout and compassion fatigue. Our study examines Canadian oncologists.

Burnout is a stress-related condition characterized by emotional exhaustion, negative attitudes, and dehumanization of clients [11, 12]. Workload, in particular, is a highly consistent predictor of burnout, both in oncology [13] and in other professions [12]. Studies of physicians have linked a range of objective and subjective workload measures to burnout, including weekly work hours [14–17]; number of nights on call [17]; perceptions of work as “overwhelming” [18]; and overall perceived job stress [19].

Compassion fatigue is rarely examined among physicians, even in specialties such as oncology that involve frequent exposure to the emotional toll of death and dying. Compassion fatigue is a phenomenon typically understood to encompass both being too tired to care, and “having to forgo [one’s] sense of compassion in an effort to protect [ones]self from despair” [20]. The process of emotional exhaustion leading to an inability to function as a compassionate provider is central to Maslach’s conceptualization of burnout [11]. Few studies examine compassion fatigue among physicians, but hours worked per shift among trauma nurses [21] and physicians’ number of days on call have been linked to high compassion fatigue [8].

While burnout and compassion fatigue may appear to be similar in that caregivers suffering from either may lose the ability to care for patients, they differ in how they come about. Burnout is linked to exhaustion that comes from chronic overwork and caring for others, whereas compassion fatigue is linked to exposure to extremely or traumatically stressful events associated with patients’ pain and suffering. Burnout can be experienced without exposure to others’ trauma whereas compassion fatigue is directly linked to secondary traumatic stress [21]. Potter et al. report that oncology nurses experience compassion fatigue as a result of “repeated exposure to patients suffering the effects of trauma, such as side effects of aggressive treatments and the end stages of cancer” (p. E57) [22]. Burnout may arise from somewhat minor chronic stressors that cumulate day after day and over time become too overwhelming, whereas compassion fatigue may result from exposure to a single extremely stressful event [23].

While research has established that workload is an important predictor of burnout, studies have not fully disentangled whether subjective or objective dimensions of workload matter more, nor examined the relative importance of these factors in predicting compassion fatigue. Subjective time pressure has been conceptualized as the subjective side of role demands [24]. Among doctors, subjective time pressure at work may represent the extent to which overall work role demands exceed one’s capacity to complete them. Perceived time pressure is associated with stress [25, 26], and high time pressures at work significantly hinder the ability to detach from work at the end of the workday [27].

Research suggests that the work/non-work interface may be a critical link between oncologists’ workload and burnout [28]. According to the work-home resources model, high work demands may threaten home outcomes, such as family relationships, through a loss in personal resources [29]. In contrast to the general population, U.S. physicians’ levels of burnout have risen in recent years while their satisfaction with work-life balance has decreased [5]. Oncologists in the U.S. report the lowest levels of satisfaction with work-life balance of any medical specialty, with only 33.4% reporting satisfaction [30]. The extent to which work interferes or conflicts with home life has been found to contribute to burnout in the medical profession [15, 16, 31, 32]. Work-home interference has also been found to mediate the relationship between work hours and burnout among both Dutch and American physicians [32]. Although research has also linked work hours to compassion fatigue [21], the role of work-home conflict has not been examined in relation to compassion fatigue.

The current study considers whether time pressure at work is a significant, independent predictor of oncologists’ burnout and compassion fatigue, and whether this occurs through greater work-family conflict. Conflicts between work and family are expected to occur when perceived workload rises, creating greater feelings of time pressure, and inhibiting detachment at the end of the workday [27]. This study contributes to the literature on the mental health of oncologists by (1) looking at predictors of *compassion fatigue* in addition to burnout, (2) focusing on the role of *time pressure* at work in relation to both burnout and compassion fatigue, and (3) assessing the potential for *work-family conflict* to mediate any relationships uncovered between time pressure and burnout or compassion fatigue. We expect that subjective time pressure will be a better predictor of burnout and compassion fatigue than oncologists’ quantitative workloads (e.g. work hours, number of hours seeing patients, number of days on call). Additionally, we expect that the relationships between time pressure and burnout, and between time pressure and compassion fatigue, will be partially mediated by work-family conflict.

## Methods

In November 2013, all practicing medical (*N* = 486) and radiation (*N* = 474) oncologists in Canada, and a limited number of medical and radiation oncology residents (*N* = 125) at English-speaking medical programs were invited to complete an online or paper version of the survey. Practicing physicians’ addresses were obtained from the Canadian Medical Directory and provincial physician colleges’ web directories. Residents’ contact information was obtained from resident program directors. All potential participants were initially sent both postal and electronic invitations. Two follow-up reminder emails were sent at 4 and 6 weeks after the initial invitation. The response rates were 35% for the oncologists and 33% for the residents. Comparisons made to the Canadian Medical Association’s 2013 national statistics on medical and radiation oncologists’ gender shows that the distributions in our sample do not differ significantly from the national ones (available from authors). The questionnaire was strictly confidential and anonymous, and the study received ethics approval.

Of the 379 oncologists and residents responding to the survey, a total of 67 cases with missing data on variables of interest (including 40 cases with missing data on number of children) were dropped from the analyses. The 312 oncologists in the present sample are represented by 131 women and 181 men. Most (90%) were married or cohabitating and 66% had children living at home. They had been practicing medicine for about 16 years on average, although the residents could be in their first year of practice while others had practiced for more than 40 years. The majority (72%) worked in an academic setting and most (46%) worked 41–50 h on-site when not on call. In this sample, mean levels of burnout and compassion fatigue are relatively low (2.92 and 2.40, respectively, on a 5 point scale), while time pressure at work and work-family conflict are somewhat higher (3.77 and 3.25, respectively) (Table 1).

**Table 1:** Summary Statistics for All Variables

	mean	SD	min	max
Burnout	2.92	0.85	1.00	5.00
Compassion fatigue	2.40	0.74	1.00	4.67
Subjective time pressure	3.77	0.83	1.00	5.00
Work-family conflict	3.25	0.92	1.00	5.00
Female (1 = yes/0 = no)	0.42	0.49	0.00	1.00
Work hours per week	3.32	0.90	1.00	6.00
Hours per week seeing patients	2.75	0.66	1.00	5.00
Hours working from home per week	2.67	0.83	1.00	4.00
On call at least 4 days/month (1 = yes/0 = no)	0.38	0.49	0.00	1.00
Academic (vs. community hospital or private practice)	0.72	0.45	0.00	1.00
Medical oncology specialty (vs. radiation oncology)	0.53	0.50	0.00	1.00
Years of Experience	16.11	9.88	0.00	46.00
Treats breast cancer (1 = yes/0 = no)	0.58	0.49	0.00	1.00
Treats sarcoma (1 = yes/0 = no)	0.23	0.42	0.00	1.00
Number of children	1.42	1.30	0.00	7.00
Married or cohabitating (1 = yes/0 = no)	0.90	0.30	0.00	1.00

N = 312

### Measures

To reduce survey length and enhance response rates [33] we used shortened versions of established scales in some cases. Unless otherwise indicated, the response categories included: never (coded 1), not very often (coded 2), sometimes (coded 3), often (coded 4), and most of the time (coded 5). The question wording for each measure is available in Additional file 1.

*Burnout* was operationalized as emotional exhaustion, considered the most essential dimension of burnout, and was measured by five items from Barnett, Brennan and Gareis’s [34] revised version of the Emotional Exhaustion subscale from the Maslach Burnout Inventory’s (MBI) General Survey (α = .89).

*Compassion Fatigue* was measured by three items adapted from Stamm’s [35] ProQOL Compassion Fatigue sub-scale of secondary traumatic stress that tap how frequently respondents experienced emotional pain and stress as a result of their patients’ suffering (α = .78).

*Subjective Time Pressure at work* was measured by four items adapted from Marks and MacDermid [36] that tap how frequently respondents experience having too much to do at work and not enough time in which to do it (α = .89).

*Quantitative workload* is measured by four variables that include: (1) typical weekly *work hours* spent at work when not on call; (2) weekly hours spent on *work brought home* when not on call; (3) weekly *hours with patients* in a typical week when not on call; and (4) if they have at least 4 *days on call* in a typical month.

*Work-Family Conflict* was measured by three items adapted from Netemeyer, Boles and McMurrian [37] that assess how frequently respondents reported work getting in the way of family (α = .92).

Personal and work-related variables found relevant to burnout, time pressure, or job resources in other studies were included as controls, including: sex, years of experience, oncology specialty (medical or radiation), work setting (university-affiliated academic center, versus community hospital or private practice), marital status, and number of children. We also coded for treating breast cancer, as physicians perceive women as experiencing greater pain [8], which could add to job strain. Treating rare forms of cancer such as sarcoma, on the other hand, may create beneficial challenge and variation in daily work [38], and is included as a control.

### Plan of analyses

Our analysis proceeds in three steps. As an initial step (1), we explore the predictors of time pressure at work. This allows us to understand how subjective time pressure may covary with quantitative workload and other key factors. Next (2), we examine how subjective time pressure at work, net of quantitative workload and various controls, predicts both burnout and compassion fatigue. Finally (3), we assess the extent to which work-family conflict mediates any relationship between time pressure at work and burnout or compassion fatigue. Zero-order correlations among all variables included in the analyses are provided in Additional file 2.

## Results

### Predictors of time pressure

We first investigate the relationships between the personal control variables and quantitative workload with perceptions of time pressure. Work environment (academic vs. non-academic) was not a significant predictor of time pressure, and reduced the adjusted R-squared measure, so it was not included as a control. Table 2 confirms that quantitative workload is predictive of subjective time pressure at work. Specifically, weekly work hours spent at work and at home are significantly related to greater time pressure at work (*p* < .001), although hours seeing patients and days on call per month are not significant predictors at *p* < .05. We also find that women, medical oncologists (as compared to radiation oncologists), and respondents with fewer years of oncology experience, report greater time pressure at work.

**Table 2:** OLS Predictors of Subjective Time Pressure at Work (*N* = 312)

	Coefficients
Female	0.191^*^
Work hours per week	0.274^***^
Hours per week seeing patients	0.024
Hours spent per week on work at home	0.235^***^
On call at least 4 days/month	− 0.172^+^
Medical specialty (vs. radiation)	0.263^**^
Years of experience	− 0.009^*^
Constant	2.158^***^
R-squared	0.220

^***^ *p* < 0.001, ^**^ *p* < 0.01, ^*^ *p* < 0.05, ^+^ *p* < 0.10

### Predictors of burnout and compassion fatigue

Our key analyses are presented in Tables 3 and 4. We first examine the predictors of burnout and compassion fatigue (Table 3). The models in Table 3 assess the predictive roles of both subjective time pressure and quantitative workload, net of gender (model 1), before examining additional controls for occupational and family characteristics. A number of non-significant quantitative workload variables were trimmed from model 2.

**Table 3:** OLS Estimates Predicting Mental Health Among Oncologists (N = 312)

	Burnout		Compassion Fatigue	
	Model 1	Model 2	Model 1	Model 2
Female	0.230^*^	0.140	0.161+	0.146+
Subjective time pressure	0.367^***^	0.380^***^	0.128^*^	0.130^*^
Work hours per week	− 0.106^*^	− 0.098+	− 0.076	− 0.077
Hours spent seeing patients	0.055		− 0.039	
Hours on work brought home	0.035		0.004	
On call at least 4 days/month	− 0.081		0.044	
Academic work setting		− 0.202^*^		− 0.087
Medical specialty (vs. radiation)		0.116		0.116
Years of experience		− 0.003		0.002
Treats breast cancer		0.231^*^		0.125
Treats sarcoma		− 0.307^**^		− 0.283^**^
Number of kids		− 0.120^***^		− 0.056+
Married or cohabitating		0.090		0.367^**^
Constant	1.574^***^	1.920^***^	2.215^***^	1.809^***^
R-squared	0.161	0.231	0.041	0.108

^***^ p < 0.001, ^**^ p < 0.01, ^*^ p < 0.05, ^+^ p < 0.10

**Table 4:** Examination of Work-to-Family Conflict as a Mediator of Time Pressure (N = 312)

	Burnout		Compassion Fatigue	
	Model 2	Model 3	Model 2	Model 3
Female	0.140	0.118	0.146^+^	0.137
Subjective time pressure	0.380^***^	0.100	0.130^*^	0.014
Work hours per week	− 0.098^+^	− 0.123^*^	− 0.077	− 0.087^+^
Academic work setting	− 0.202^*^	− 0.135	− 0.087	− 0.059
Medical specialty (vs. radiation)	0.116	0.121	0.116	0.118
Years of experience	− 0.003	− 0.001	0.002	0.003
Treats breast cancer	0.231^*^	0.166^+^	0.125	0.098
Treats sarcoma	− 0.307^**^	− 0.326^**^	− 0.283^**^	− 0.290^**^
Number of kids	− 0.120^***^	− 0.138^***^	− 0.056^+^	− 0.064^+^
Married or cohabitating	0.090	0.038	0.367^**^	0.346^*^
Work-family conflict		0.413^***^		0.170^**^
Constant	1.920^***^	1.744^***^	1.809^***^	1.737^***^
R-squared	0.231	0.340	0.108	0.132

^***^ p < 0.001, ^**^ p < 0.01, ^*^ p < 0.05, ^+^ p < 0.10

As shown in the analyses predicting burnout, women report higher levels of burnout, even net of subjective and objective workload (model 1). Controlling for occupational and family characteristics (model 2), however, accounts for the effect of gender. We also find that weekly work hours is negatively associated with burnout in model 1 (*p* < .10), ceteris paribus.

Our key independent variable, subjective time pressure at work, is a significant predictor of burnout, net of gender and quantitative workload in model 1. In model 2, with an alternate set of control variables representing family and occupational characteristics, time pressure at work continues to be a highly significant predictor of burnout (*p* < .001), with no decrease in effect size.

Family and occupational characteristics are also independent predictors of burnout. Parenthood is negatively associated with burnout. Working in an academic setting, and treating sarcoma, are both negatively associated with burnout, while treating breast cancer is positively associated with burnout.

The next set of models in Table 3 show the predictors of compassion fatigue. Here, subjective time pressure is a significant predictor of compassion fatigue at *p* < .05 in both models 1 and 2. Women also experience higher compassion fatigue scores in both models 1 and 2, although these associations are significant only at *p* < .10. Quantitative workload is not a significant independent predictor of compassion fatigue in either model.

In model 2, similar to burnout, treating sarcoma is significantly associated with lower compassion fatigue scores. Parenthood is also associated with lower compassion fatigue scores, although this is significant only at p < .10. Surprisingly, we also observe a positive, significant relationship between marriage and compassion fatigue. Overall, the models predicting compassion fatigue explain less of the total variation (i.e., 4% and 11%) than those predicting burnout (16% and 23%).

### Work-family conflict as a mediating variable

Our last set of analyses examine whether work-family conflict mediates the predictive role of time pressure shown in the previous set of results. Table 4 displays the predictors of burnout and compassion fatigue shown previously in Table 3, model 2. Additionally, a model including a measure of work-family conflict is included for both burnout and compassion fatigue (model 3).

As shown in Table 4, including work-family conflict in the models predicting burnout and compassion fatigue accounts completely for the effect of time pressure at work (model 3 for each outcome). When work-family conflict is included as a predictor, its effect is positive and significant, while the effect of time pressure at work is no longer significant, and its coefficient decreases markedly.

We further investigated the seeming mediation effects shown in Table 4 by bootstrapping the indirect effects using Stata, which performs a Sobel test. These supplemental analyses confirmed that work-family conflict fully mediates the effect of time pressure at work on burnout and compassion fatigue. This type of mediation is “indirect only;” no direct effect of time pressure at work remains when the mediating effect of work-family conflict is included in the models [39]. Again, similar to the analyses presented in Table 3, the proportion of overall variation explained in these models is much greater for burnout than for compassion fatigue.

## Discussion

While burnout has been studied extensively among physicians, research has less often examined physicians’ experiences of compassion fatigue, or whether time pressure at work might contribute to these mental health outcomes independently of quantitative workload. This study of oncologists shows that subjective perception of time pressure at work is a key predictor of burnout and compassion fatigue above and beyond reports of quantitative workload or other aspects of the work environment. The phenomenon of time deepening, or needing to get more done in a given period of time, is an important predictor of oncologists’ mental health that should be considered in addition to the total number of hours worked, or the number of hours spent on particular tasks.

We also find that the relationship between time pressure at work and oncologists’ mental health is fully mediated by work-family conflict. This suggests that time pressure in the work environment may affect mental health by contributing to conflicts at home. Some research indicates that time pressure at work inhibits disengaging from work psychologically after work, which may lead to role conflicts [27]. Other research suggests that this may be part of a larger “loss spiral,” in which time pressure leads to worsening work-family conflict and mental health, work-family conflict leads to worsening time pressure and mental health, and poor mental health contributes to worsening time pressure and work-family conflict [40]. Our findings present only a snapshot of associations, and cannot assess these complex relationships. However, because the work environment is an exogenous set of arrangements that can be optimized to promote mental health and reduce work-family conflict, research on outcomes of work environments can be translated into best practices for mental health. The results of this study indicate that addressing time pressure in the work environment may be a way to reduce work-family conflict and improve mental health among oncologists. Although our data are cross-sectional and we are unable to show a causal process, our findings are supportive of a link between time pressure, work-family conflict, and oncologists’ mental health. Future research should examine these issues longitudinally among medical practitioners to address issues of selection and to control for time stable characteristics, such as ongoing mental health problems that might contribute to elevated perceptions of time pressure and conflict.

We also find that more overall variation is explained in our models of burnout than compassion fatigue, raising questions about what unmeasured characteristics or experiences might also contribute to compassion fatigue. While subjective experiences of time pressure and work-family conflict appear particularly important, there is clearly a need to develop more comprehensive models of compassion fatigue in order to better understand the work-related factors responsible. One approach might be to include factors reflecting workers’ exposure to traumatic events, coping strategies, personal and environmental characteristics and various stress reactions, which was beyond the scope of this study [21]. A more comprehensive model not only offers the possibility of better understanding compassion fatigue but also of being able to identify the key factors responsible in order to enable health care workers and their employing organizations to take necessary steps to prevent or respond to the impending development of compassion fatigue.

Because of the strong relationship between quantitative workload and time pressure in the work environment, our research is supportive of prior recommendations to reduce clinical work hours and patient load as a strategy to reduce oncologist burnout [41]. However, because we find that work pressure is a stronger predictor of burnout and compassion fatigue than quantitative workload, a more comprehensive strategy that considers work hours and patient load in tandem with other factors associated with work pressure and work-family conflict may be more effective. In particular, a workplace culture that supports family-friendly resources, particularly scheduling control/flexibility, has been found to be effective in reducing work-family conflicts [42]. Research has shown that family-supportive workplace cultures are associated with reduced work-family conflicts, whereas simply having family-supportive policies, which may or may not be supported or encouraged, do not [43]. A supportive work-family culture may be particularly important for physicians who often place their patients’ health and wellbeing above their own, and who work within the broader culture of medicine that tends to deter doctors from taking care of themselves [4]. As physician mental health is an important factor in the quality of patient care, supportive work cultures would benefit both oncologists and their patients.

In addition to the cross-sectional nature of our data, and the small sample size, this study is limited by the survey measures used. Our mental health measures cannot assess prevalence of emotional exhaustion or compassion fatigue, only a relative higher or lower score for each respondent. Although our shortened scales are sufficient for examining the relevance of certain predictors, studies wishing to assess prevalence would need to use the complete and standardized measures of burnout and compassion fatigue (e.g., the 22 item Maslach Burnout Inventory and the 30 item Professional Quality of Life Scale). In doing so, burnout and compassion fatigue prevalence scores can be compared to other occupations, as well as across other individual characteristics (e.g., gender, age, years in the field) that may be helpful for decision makers attempting to managing stress and trauma in the workplace.

## Conclusions

In closing, this study shows that oncologists’ subjective perception of time pressure at work is a key predictor of their burnout and compassion fatigue, and closely linked to work-family conflict. It is therefore important to consider the subjective aspects of workload in addition to quantitative workload. Workplace cultures allowing greater flexibility to access personal time should be investigated in the oncology setting.

## Additional files

Additional file 1: Abridged Burnout Survey for Canadian Oncologists. Contains the abridged Burnout Survey for Canadian Oncologists that includes the question wording and format for all of the measures used in this paper. (DOCX 23 kb)

Additional file 2: Zero-Order Correlation Matrix of Dependent, Independent, Covarying, and Mediating Variables. Contains the zero-order correlation matrix for all variables included in the multivariate analyses presented in this paper. (DOCX 15 kb)

## References

[1] Schernhammer ES, Colditz GA (2004). Suicide rates among physicians: a quantitative and gender assessment (meta-analysis). Am J Psychiatry.

[2] Dyrbye LN (2008). Burnout and suicidal ideation among U.S. medical students. Ann Intern Med.

[3] Shanafelt TD (2011). Special report: suicidal ideation among American surgeons. Arch Surg.

[4] Wallace JE, Lemaire JB, Ghali WA (2009). Physician wellness: a missing quality indicator. Lancet.

[5] Shanafelt TD (2015). Changes in burnout and satisfaction with work-life balance in physicians and the general US working population between 2011 and 2014. Mayo Clin Proc.

[6] Le Blanc PM (2001). Emotional job demands and burnout among oncology care providers. Anxiety Stress Coping.

[7] Grunfeld E (2000). Cancer care workers in Ontario: prevalence of burnout, job stress and job satisfaction. CMAJ.

[8] Gleichgerrcht E, Decety J (2014). The relationship between different facets of empathy, pain perception and compassion fatigue among physicians. Front Behav Neurosci.

[9] Grunfeld E (2005). Job stress and job satisfaction of cancer care workers. Psycho-Oncology.

[10] Granek L (2012). Nature and impact of grief over patient loss on oncologists’ personal and professional lives. Arch Intern Med.

[11] Maslach C (1976). Burned-out. Human Behavior.

[12] Maslach C, Schaufeli WB, Leiter MP (2001). Job burnout. Annu Rev Psychol.

[13] Sherman AC (2006). Caregiver stress and burnout in an oncology unit. Palliat Support Care.

[14] Balch CM (2010). Surgeon distress as calibrated by hours worked and nights on call. J Am Coll Surg.

[15] Dyrbye LN (2011). Relationship between work-home conflicts and burnout among American surgeons: a comparison by sex. Arch Surg.

[16] Dyrbye LN (2011). Work/home conflict and burnout among academic internal medicine physicians. Arch Intern Med.

[17] Shanafelt TD (2009). Burnout and career satisfaction among American surgeons. Ann Surg.

[18] Campbell DA (2001). Burnout among American surgeons. Surgery.

[19] Elit L (2004). Job satisfaction, stress, and burnout among Canadian gynecologic oncologists. Gynecol Oncol.

[20] Berzoff J, Kita E (2010). Compassion fatigue and countertransference: two different concepts. Clin Soc Work J.

[21] Hinderer KA (2014). Burnout, compassion fatigue, compassion satisfaction, and secondary traumatic stress in trauma nurses. J Trauma Nurs.

[22] Potter P (2010). Compassion fatigue and burnout: prevalence among oncology nurses. Clin J Oncol Nurs.

[23] Kumar S (2016). Burnout and doctors: prevalence, prevention and intervention. Healthcare.

[24] Roxburgh S (2004). ‘There just aren’t enough hours in the day’: the mental health consequences of time pressure. J Health Soc Behav.

[25] Williams ES (2002). Physician, practice, and patient characteristics related to primary care physician physical and mental health: results from the physician Worklife study. Health Serv Res.

[26] Kleiner S (2014). Subjective time pressure: general or domain specific?. Soc Sci Res.

[27] Sonnentag S, Bayer UV (2005). Switching off mentally: predictors and consequences of psychological detachment from work during off-job time. J Occup Health Psychol.

[28] Whippen DA, Canellos GP (1991). Burnout syndrome in the practice of oncology: results of a random survey of 1,000 oncologists. J Clin Oncol.

[29] ten Brummelhuis LL, Bakker AB (2012). A resource perspective on the work-home interface: the work-home resources model. Am Psychol.

[30] Shanafelt TD (2014). Satisfaction with work-life balance and the career and retirement plans of US oncologists. J Clin Oncol.

[31] Geurts S, Rutte C, Peeters M (1999). Antecedents and consequences of work-home interference among medical residents. Soc Sci Med.

[32] Linzer M (2001). Predicting and preventing physician burnout: results from the United States and the Netherlands. Am J Med.

[33] Cummings SM, Savitz LA, Konrad TR (2001). Reported response rates to mailed physician questionnaires. Health Serv Res.

[34] Barnett RC, Brennan RT, Gareis KC (1999). A closer look at the measurement of Burnout1. J Appl Biobehav Res.

[35] Stamm, B.H. Professional quality of life scale (ProQOL) version 5. 2009 [cited 2015; Available from: http://www.proqol.org/uploads/ProQOL_5_English.pdf.

[36] Marks SR, MacDermid SM. Multiple roles and the self: a theory of role balance. J Marriage Fam. 1996:417–32.

[37] Netemeyer RG, Boles JS, McMurrian R (1996). Development and validation of work–family conflict and family–work conflict scales. J Appl Psychol.

[38] Bakker AB, Demerouti E (2007). The job demands-resources model: state of the art. J Manag Psychol.

[39] Zhao X, Lynch JG, Chen Q (2010). Reconsidering Baron and Kenny: myths and truths about mediation analysis. J Consum Res.

[40] Demerouti E, Bakker AB, Bulters AJ (2004). The loss spiral of work pressure, work–home interference and exhaustion: reciprocal relations in a three-wave study. J Vocat Behav.

[41] Shanafelt T, Dyrbye L (2012). Oncologist burnout: causes, consequences, and responses. J Clin Oncol.

[42] Kelly EL, Moen P, Tranby E (2011). Changing workplaces to reduce work-family conflict schedule control in a white-collar organization. Am Sociol Rev.

[43] Mennino SF, Rubin BA, Brayfield A (2005). Home-to-Job and Job-toHome Spillover: the impact of company policies and workplace culture. Sociol Q.

