# Supplementary material for: Oncologist burnout and compassion fatigue: investigating time pressure at work as a predictor and the mediating role of work-family conflict
Source: BMC Health Serv Res. 2017 Sep 11;17:639. doi: 10.1186/s12913-017-2581-9 (PMC5594602; doi:10.1186/s12913-017-2581-9)
Supplement: Supplementary file 1 — Abridged Burnout Survey for Canadian Oncologists. Contains the abridged Burnout Survey for Canadian Oncologists that includes the question wording and format for all of the measures used in this paper. (DOCX 23 kb) [file 12913_2017_2581_MOESM1_ESM.docx]

Additional File 1: Abridged Burnout Survey for Canadian Oncologists

We're conducting a study exploring burnout and coping among Canadian oncologists. **Please fill in the response that best describes your current work situation.**

Which best describes your current position:

☐ Medical oncologist ☐ Radiation oncologist
☐ Resident/fellow in medical oncology ☐ Resident/fellow in radiation oncology

Please indicate the year that you started Oncology Training (e.g., 2001) _____

What cancer population(s) do you currently provide care to (please check all that apply):

☐ Breast ☐ Gastrointestinal
☐ Prostate ☐ CNS
☐ Lung ☐ Head and neck cancer
☐ Colorectal ☐ Hematological malignancies
☐ Gynecological ☐ Skin
☐ All cancer populations ☐ Genitourinary
☐ Sarcoma ☐ Phase I

What type of institution are you currently practicing in?

☐ Tertiary University Affiliated Academic Centre (e.g., BCCA Vancouver, Princess Margaret Hospital)
☐ Community Cancer Centre (e.g., BCCA Fraser Valley, Carlo Fidani Peel Regional Cancer Centre)
☐ Community Hospital and/or Private Practice

In an average **week**, when you're not on call, how many hours do you spend at work **per week** on site (eg. clinic, office, laboratory, hospital)?

☐ 0 hours ☐ 1-20 hours ☐ 21-40 hours ☐ 41-50 hours ☐ 51-60 hours ☐ 61-70 hours ☐ >70 hours

In an average **week**, when you're not on call, how many **hours** do you spend:

- performing **work** tasks at **home** (eg checking email, research)? ☐ 0 hours ☐ 1-5hours ☐ 6-10 hours ☐ 11 or more hours per week
- seeing patients? ☐ 0 h ☐ 1-20 hours ☐ 21-40 hours ☐ 41-50 hours ☐ 51-60 hours☐ 61-70 hours ☐ >70 hours per week

What is your average frequency of **call** per month?

☐ 0-3 ☐ 4-7 ☐ 8-12 ☐ > 12

The next section asks about the demands of your job. Practicing medicine can be extremely satisfying and demanding at time. Please indicate the extent to which the following statements best describe your current work attitudes or experiences.

	Never	Not very often	Sometimes	Often	Most of the time	N/A
I do not have enough time to get everything done.	☐	☐	☐	☐	☐	☐
I need more hours in the day to do all of the things that are expected of me.	☐	☐	☐	☐	☐	☐
I often overextend myself in order to finish everything I have to do.	☐	☐	☐	☐	☐	☐
I often feel rushed in my work.	☐	☐	☐	☐	☐	☐
The demands of my work interfere with my home and personal life.	☐	☐	☐	☐	☐	☐
Things I want to do in my personal or family life do not get done because of the demands of my work.	☐	☐	☐	☐	☐	☐
The amount of time my work takes up makes it difficult for me to fulfill my family responsibilities.	☐	☐	☐	☐	☐	☐

When you help people you have direct contact with their lives. Sometimes your compassion for those you help can affect you in positive and negative ways. Consider each of the following statements and how frequently you have experienced these things in the last 30 days.

	Never	Not very often	Sometimes	Often	Most of the time	N/A
I feel depressed because of the stressful experiences of my patients.						
I feel as though I experience the emotional pain of my patients.						
I feel I may be affected by the traumatic stress of those I help in my work.						
I feel emotionally drained from my work.						
I feel used up at the end of the work day.						
I feel tired when I get up and have to face another day of work.						
I feel that working all day is really a strain for me.						
I feel burned out from my work.						

The next set of questions is intended to collect information on your background characteristics. Please keep in mind that the results will be used in aggregate form only.

What is your age? ☐ 25-35 ☐ 36-50 ☐ 51-60 ☐ 61+

What is your sex? ☐ Male ☐ Female

Which of the following best describes your present situation? (check one only)

☐ Single/Never Married ☐ Cohabitating/Common Law ☐ Married

☐ Separated/Divorced ☐ Widowed

How many children are currently living at home with you in the following age groups?

___ under 6 years of age ___ 6-12 years of age ___ 13-18 years of age ___ 18 years of age or older

If there is anything else you would like to share about your work experiences, please write your comments below.

Thank you for your time and effort in completing this questionnaire.
Your contributions to this study is very much appreciated.
