# Supplementary material for: Oncologist burnout and compassion fatigue: investigating time pressure at work as a predictor and the mediating role of work-family conflict
Source: BMC Health Serv Res. 2017 Sep 11;17:639. doi: 10.1186/s12913-017-2581-9 (PMC5594602; doi:10.1186/s12913-017-2581-9)
Supplement: Supplementary file 2 — Zero-Order Correlation Matrix of Dependent, Independent, Covarying, and Mediating Variables. Contains the zero-order correlation matrix for all variables included in the multivariate analyses presented in this paper. (DOCX 15 kb) [file 12913_2017_2581_MOESM2_ESM.docx]

Additional File 2. Correlation Matrix of Dependent, Independent, Covarying, and Mediating Variables

	Burnout	CF	TP	WFC	F	Hours	Hrs Pts	Wk home	On call	Academic	Medical	Experience	Breast	Sarcoma	Kids	Mar/Cohab
Burnout	1															
Compassion Fatigue	0.5506*	1														
Time Pressure	0.3550*	0.1286*	1													
Work/Family Conflict	0.4847*	0.2028*	0.6439*	1												
Sex	0.1819*	0.1311*	0.1244*	0.1322*	1											
Hours Worked / Week	-0.0087	-0.064	0.3047*	0.2295*	-0.0837	1										
Hours Seeing Patients	0.0424	-0.0455	0.0641	0.0116	-0.0488	0.111	1									
Work at Home (Hours)	0.1403*	0.0404	0.3004*	0.2722*	0.0938	0.1222*	0.0981	1								
On call 4+ days/month	-0.0325	-0.0306	-0.0107	0.0321	0.1336*	0.0941	0.1670*	0.0926	1							
Academic Setting	-0.0856	-0.0485	0.0763	-0.0438	0.0121	0.0518	-0.1447*	0.0313	-0.3085*	1						
Medical Specialty	0.1646*	0.1218*	0.1883*	0.1262*	0.076	-0.0066	0.0977	0.1151*	0.0416	-0.1185*	1					
Experience (Years)	-0.0556	0.0531	-0.1274*	-0.1665*	-0.1502*	0.0162	0.0353	-0.0882	-0.1401*	-0.085	0.0068	1				
Treats Breast Cancer	0.1912*	0.0945	0.0319	0.1271*	0.3107*	-0.0872	0.0562	0.1474*	0.2187*	-0.1905*	0.0742	-0.1345*	1			
Treats Sarcoma	-0.0518	-0.1451*	0.0611	0.0864	0.0617	0.028	0.067	0.1452*	0.2394*	-0.0367	0.041	-0.2991*	0.2193*	1		
Number of Kids	-0.1489*	-0.0435	-0.0017	0.0637	-0.0359	-0.0565	-0.0072	-0.1497*	0.0051	-0.1059	-0.0206	-0.0362	-0.0971	-0.1817*	1	
Married/Cohabitating	-0.0083	0.1424*	-0.0039	0.0265	-0.0786	0.0114	0.0661	0.0056	0.0067	0.0515	0.0005	0.0776	-0.1164*	-0.1910*	0.1980*	1

* p<.05
